# PW03-016 – Blau prospective cohort study: articular outcomes

**DOI:** 10.1186/1546-0096-11-S1-A242

**Published:** 2013-11-08

**Authors:** CD Rose, R Cimaz, C Thomee, R Khubchandani, G Espada, R Russo, M Harjacek, B Bader-Meunier, P Brissaud, N Wulffraat, S Vastert, R Merino, A Naranjo-Hernandez, S Oliveira-Knupp, F Mackensen, J Arostegui, J Anton, J Fernandez-Martin, C Wouters

**Affiliations:** 1 Pediatrics, Nemours A. I. Dupont Hospital For Children, Wilmington, USA; 2 Universita degli Studi de Firenze, Firenze, Italy; 3 Centre Hospitalier de Luxemburg, Luxembourg, Luxembourg; 4 Jaslok Hospital, Mumbai, India; 5 Universidad de Buenos Aires, Buenos Aires, Argentina; 6 University of Buenos Aires, Buenos Aires, Argentina; 7 University of Zagreb, Zagreb, Croatia; 8 Hospital Necker Enfants malades, Paris, France; 9 None, Neully/Seine, France; 10 UMC Utrecht, Utrecht, Netherlands; 11 Hospital La Paz, Madrid, Spain; 12 Hospital Dr Negrin, Gran Canarias, Spain; 13 Universidad Federal do Rio de Janeiro, Rio de Janeiro, Brazil; 14 Universitat Klinikum Heidelberg, Heidelberg, Germany; 15 Universidad de Barcelona, Barcelona; 16 Hospital do Meixoeiro, Vigo, Spain; 17 Katholieke Universiteit Leuven, Leuven, Belgium

## Introduction

Blau syndrome is an autosomal dominant monogenic granulomatous disease associated with gain of function mutations at or near the NACHT domain of NOD2; it is the only form of granulomatous arthritis with a known gene mutation. Although its phenotype has been amply described as a triad of arthritis, uveitis and dermatitis in case series and retrospective cohorts, prospective studies on natural history and outcome have not been done.

## Objectives

To prospectively study in detail the phenotypic characteristics, functional articular and visual outcomes and radiographic progression of joint disease in patients with BS. Secondary goals are to investigate biomarkers of disease activity as well as to explore relevant pathogenic pathways and candidates for therapeutic targeting.

## Methods

Participating centers of an international registry were invited to enroll patients with documented NOD2 mutation after IRB approval. This 3 year prospective study consists of one baseline and 3 yearly visits comprising a comprehensive clinical evaluation, functional assessment (CHAQ/HAQ), visual analogue scales, full ophthalmologic assessment and wrists/hand radiographs at baseline and at last evaluation. Poznansky and Sharp scores were utilized to analyze pediatric and adult X-rays respectively. Blood sampling was performed for follow up and exploratory for biomarkers. Drug therapy was recorded. Coded data were kept in a secured database at the coordinating center.

## Results

We are reporting here baseline articular and functional data of the first 25 recruited patients. F: 8; M: 17. Ages:0-54 years;50% 0-15. More than half carried substitution R334W or R334Q. Onset of joint disease was 33 months (3-156). At evaluation arthritis duration was 15.7 yrs (1-53). Mean active joint count was 7 (0-24). Mean CHAQ/HAQ 0.42 (0-2). VAS-p 1.78 (0-8) and VAS-g 2.06 (0-8). A subgroup of patients with long duration (20-50 years) showed a mean joint count of 11.4 (1-24), HAQ of 0.9 (0-2), VAS-p of 4.9 (0-8), VAS-g of 4.1 (0-8). 11/25 required daily systemic prednisone with methotrexate and/or biologics. Significant destructive radiographic changes were documented over time. 50% of the entire group showed extra-triad manifestations with lymphadenopathy, fever, erythema nodosum and hypertension the most common.

## Conclusion

This first prospective study on the natural history of BS demonstrates a relentlessly active and destructive articular involvement with significant functional morbidity, exhibiting high levels of disability and disease activity even after years of multiple therapies.

## Disclosure of interest

None declared

